# Association of frailty with dementia and the impact of frailty on antihypertensive treatment protection against dementia in older hypertensive adults

**DOI:** 10.1093/ageing/afag037

**Published:** 2026-02-25

**Authors:** Linan Chen, Shoujiang You, Nicole Ee, Kenneth Rockwood, David D Ward, Ruth E Hubbard, Mark Woodward, Oisin Fitzgerald, Ruirui Wang, Yijie Gao, Jeff D Williamson, Craig S Anderson, Katie Harris, Xiaoying Chen, Ruth Peters

**Affiliations:** The George Institute for Global Health, University of New South Wales Faculty of Medicine, Sydney, New South Wales, Australia; The George Institute for Global Health, University of New South Wales Faculty of Medicine, Sydney, New South Wales, Australia; Department of Neurology and Clinical Research Center of Neurological Disease, Second Affiliated Hospital of Soochow University, Suzhou, Jiangsu, China; The George Institute for Global Health, University of New South Wales Faculty of Medicine, Sydney, New South Wales, Australia; Division of Neurology, Dalhousie University Department of Medicine, Halifax, Nova Scotia, Canada; Division of Geriatric Medicine, Dalhousie University Department of Medicine, Halifax, Nova Scotia, Canada; Frailty and Elder Care Network, Halifax, Nova Scotia, Canada; Australian Frailty Network, Centre for Health Services Research, The University of Queensland Faculty of Health and Behavioural Sciences, Brisbane, Queensland, Australia; Princess Alexandra Hospital, Woolloongabba, Queensland, Australia; Australian Frailty Network, Centre for Health Services Research, The University of Queensland Faculty of Health and Behavioural Sciences, Brisbane, Queensland, Australia; Princess Alexandra Hospital, Woolloongabba, Queensland, Australia; The George Institute for Global Health, University of New South Wales Faculty of Medicine, Sydney, New South Wales, Australia; The George Institute for Global Health, Imperial College London School of Public Health, London, England, UK; National Perinatal Epidemiology and Statistics Unit, University of New South Wales, Sydney, New South Wales, Australia; The George Institute for Global Health, University of New South Wales Faculty of Medicine, Sydney, New South Wales, Australia; Department of Epidemiology, Suzhou Medical College of Soochow University School of Public Health, Suzhou, Jiangsu, China; The George Institute for Global Health, University of New South Wales Faculty of Medicine, Sydney, New South Wales, Australia; Section of Gerontology and Geriatric Medicine, Wake Forest University Department of Internal Medicine, Winston-Salem, NC, USA; The George Institute for Global Health, University of New South Wales Faculty of Medicine, Sydney, New South Wales, Australia; Fudan University Institute of Science and Technology for Brain-inspired Intelligence, Shanghai, China; The George Institute for Global Health China, Beijing, China; Neurology Department, Royal Prince Alfred Hospital, Camperdown, New South Wales, Australia; The George Institute for Global Health, University of New South Wales Faculty of Medicine, Sydney, New South Wales, Australia; The George Institute for Global Health, University of New South Wales Faculty of Medicine, Sydney, New South Wales, Australia; The George Institute for Global Health, University of New South Wales Faculty of Medicine, Sydney, New South Wales, Australia

**Keywords:** frailty, antihypertensive agents, dementia, aged, clinical trial, older people

## Abstract

**Background:**

The relationship between frailty and dementia risk in hypertensive patients remains unclear, as does the impact of frailty on the effectiveness of antihypertensive treatment in preventing dementia.

**Methods:**

Using data from the Systolic Hypertension in the Elderly Program trial, a baseline frailty index (FI) including 55 health deficits was constructed. Multinomial regression models were used to examine the association between frailty and dementia, as well as to examine whether the impact of antihypertensive treatment on the risk of dementia was modified by baseline FI.

**Results:**

A total of 4692 participants (mean age: 72.1 years, 56.8% female) were included with a median (inter-quartile interval) FI of 0.127 (0.091–0.173). During a median follow-up of 4.4 years, 81 dementia cases occurred. Each SD (0.061) increase in FI was associated with a 33% higher risk of dementia [odds ratio (OR): 1.33, 95% confidence interval (CI): 1.08–1.64], after adjusting for age, sex, race, education and treatment group. This association differed between the active treatment group (OR: 1.57; 95% CI: 1.16–2.11) and the placebo group (OR: 1.17; 95% CI: 0.87–1.56), with a *P* for interaction = .049. Correspondingly, in the lowest FI quarter, antihypertensive treatment reduced dementia risk (OR: 0.19, 95% CI: 0.04–0.94), an effect not observed in higher FI quarters (interaction *P* = .058).

**Conclusion:**

In patients with isolated systolic hypertension, frailty may serve as a risk factor for dementia, and elevated levels of frailty may attenuate the effectiveness of antihypertensive treatment in reducing dementia risk.

## Graphical Abstract

**Graphical Abstract:**
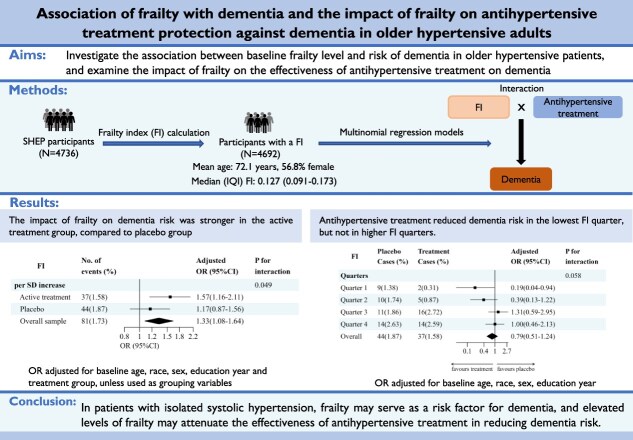

## Key Points

- Higher frailty was linked to increased dementia risk in older adults with systolic hypertension.
- Frailty’s impact on dementia was stronger in treated vs untreated hypertensive participants.
- Antihypertensive treatment reduced dementia risk more in low-frailty than high-frailty adults.
- In high-frailty patients, antihypertensive therapy alone may not suffice to reduce dementia risk.

## Introduction

Hypertension is a widely recognised risk factor for dementia [1]. Meta-analyses of randomised clinical trials of blood pressure (BP) control have shown that antihypertensive medications can reduce the risk of developing dementia in hypertensive patients by 7% to 13% [2, 3]. Frailty, a syndrome characterized by functional decline across multiple physiological systems coupled with increased susceptibility to stressors [4], has been incorporated into clinical guidelines for the management of elevated BP and hypertension as a key factor in determining BP targets and selecting antihypertensive medications [5].

As an important risk factor affecting both dementia [6–8] and hypertension [9–11], frailty may influence the relationship between BP levels and dementia risk. A large-scale observational study involving 804 024 participants aged 66 years found that higher systolic blood pressure (SBP) was linked to an increased risk of dementia, independent of participant’s physical frailty status [12]. In contrast, another cross-sectional study including 8609 participants aged 50 years old and above found that hypertension was associated with better cognitive performance among frail participants aged 65 and above [13]. This discrepancy has raised concerns about the potential effectiveness of antihypertensive treatment on dementia in frailer populations. A post hoc analysis of the Systolic Blood Pressure Intervention Trial (SPRINT) found that frailty level exerted a stronger impact on increasing the risk of cognitive impairment in the intensive BP-lowering group compared to the standard treatment group. This finding highlights the need for caution when using antihypertensive treatment in frail populations, given the potential increased risk of cognitive impairment [14]. Nevertheless, research using randomised trial data, which is essential for evaluating treatment effects, remains limited in exploring the impact of frailty on the effectiveness of antihypertensive therapy in relation to dementia.

Therefore, this study aimed to (i) investigate the association between baseline frailty level and risk of dementia in older hypertensive patients and (ii) examine the impact of frailty on the effectiveness of antihypertensive treatment on incident dementia.

## Methods

### Study population

This study was a post hoc analysis of data from the Systolic Hypertension in the Elderly Program (SHEP) trial, which is a double-blind, randomised, placebo-controlled study conducted across 16 clinical centres in the United States of America. Participants were recruited between 1985 and 1988, with follow-up completed in 1990. The objectives, study design and findings of the trial have been published previously [15]. A total of 4736 participants aged 60 years or older, with a seated SBP of 160–219 mmHg and a diastolic blood pressure (DBP) of <90 mmHg, were randomly assigned to either the active treatment group (*n* = 2365) or the placebo group (*n* = 2371). Written informed consent was obtained from all participants. The trial was registered (ClinicalTrials.gov Identifier: NCT00000514).

### Frailty index

Baseline frailty levels of participants were quantified using a frailty index (FI) developed following the standardised methodology [16], the details of which are outlined in the Appendix. Briefly, 55 variables were included from the baseline data of SHEP in the FI (Appendix Table S1). To construct the FI, each variable was recoded on a scale from 0 to 1, where 0 indicated the absence of a deficit and 1 indicated its full presence. For each participant, the FI was calculated as the sum of all deficit scores divided by the number of available variables for that participant. An FI was not calculated for 44 participants missing more than 11 variables (20% of the deficits included in FI) and those participants were excluded from the analysis. The FI was categorised into quarters, with higher quarters representing greater levels of frailty.

### Outcome

The diagnostic procedures for dementia in SHEP included screening for cognitive decline using the short-Comprehensive Assessment and Referral Evaluation (short-CARE) scale [15, 17]. The short-CARE was administered every 6 months during the follow-up period. If a participant scored, at or above, the cut-off for potential dementia (≥4 out of 9), the short-CARE was reassessed at the next quarterly visit. Participants who met, or exceeded, the cut-off at two consecutive visits were referred to an independent clinician for further diagnostic evaluation based on the Diagnostic and Statistical Manual of Mental Disorders, Third Edition, Revised (DSM-III-R). The diagnosis of dementia required confirmation by the blinded SHEP coding panel, which included two neurologists. Detailed information about the short-CARE questionnaire is available in the publicly accessible manual on behavioural evaluation [18].

### Statistical analysis

#### Characteristics of participants

Baseline characteristics of participants are presented by randomised treatment groups. Continuous variables are tabulated as mean (standard deviation, SD) and categorical variables as frequencies and percentages. The Wilcoxon rank-sum test was employed to evaluate differences in the FI between randomised treatment groups.

#### Association between baseline frailty index and dementia

To account for the competing risk of death before a potential dementia diagnosis, multinomial regression models were used to estimate the associations [odds ratios (OR) with 95% confidence intervals (CI)] between baseline FI and incident dementia. Baseline FI was represented both in ordinal quarters (base = 1st quarter) and as a continuous variable (per SD increase). An unadjusted model and a model adjusted for age, sex, race, years of education and treatment group were estimated. Cubic spline regression models were used to graphically evaluate the shape of the association between FI and dementia, as well as potential nonlinear relationships.

To evaluate whether the effect of frailty on incident dementia varied by randomised treatment groups, a multinomial regression model was used, including an interaction term between the continuous FI and treatment group. Multinomial regression models were constructed using the R package *nnet* and *rms* was used to fit restricted cubic spline regression models.

#### Impact of baseline frailty levels on the efficacy of antihypertensive treatment for dementia

The impact of baseline frailty level on the randomised treatment effect was first assessed by comparing the effectiveness of antihypertensive treatment on dementia with and without adjustment for baseline FI. Next, the effect of antihypertensive treatment by quarters of FI was estimated. Multinomial regression models were established to estimate the effect (OR, 95% CI) of antihypertensive treatment on incident dementia, with all-cause death as the competing risk event, adjusting for baseline age, sex, race and years of education. An interaction term between FI quarter and treatment was then introduced to test the heterogeneity of the antihypertensive treatment effects across the different FI quarters.

To explore how the treatment effect varied across the full range of the continuous FI, we used the marginaleffects R package [19] to estimate conditional comparisons based on a multivariable logistic regression model with dementia as the outcome, including an interaction term between treatment group and continuous FI, and adjusting for age, sex, race and years of education. This approach estimated the treatment effect (log-transformed OR with 95% CI) at different levels of frailty, while holding other covariates at representative values.

#### Sensitivity analysis

To determine whether age, sex, education and baseline SBP level affected our results, we examined the impact of frailty on the risk of dementia and treatment effectiveness by baseline subgroups: age thirds (<68 years, 68–75 years, >75 years), sex (female and male), education level (<12 years (median education years) and ≥12 years) and baseline SBP thirds (<165 mmHg, 165–173 mmHg, >173 mmHg). Additionally, we conducted sensitivity analyses using alternative FI thresholds to evaluate the robustness of our findings. Specifically, participants were dichotomized according to the FI median (FI = 0.127) and an FI cut-point of 0.21 [20] and classified into three frailty categories defined as fit (FI ≤ 0.12), mild frailty (FI > 0.12–0.24), and moderate to severe frailty (FI > 0.24) [21]. We subsequently compared dementia risk and the effectiveness of antihypertensive treatment across these groupings to assess the consistency of the results. Finally, to assess whether cardiovascular-related factors included in the FI affected our findings, we reanalysed the data after excluding these factors from the FI.

A two-tailed *P* value of <.05 was considered statistically significant. All statistical analyses were performed using R (version 4.4.2, R Foundation).

## Results

### Characteristics of participants

Of the 4736 participants from SHEP, 4692 participants with available FI data were included in this study [median (inter-quartile interval (IQI)] age = 71.5 (66.8–76.7) years, 43.2% male) mean (SD) FI of 0.134 (0.061). The baseline characteristics of participants are summarised in Table 1; as expected, there were no significant imbalances between the randomised treatment groups, including FI (median: 0.127, IQI: 0.091–0.173 in both groups, Appendix Figure S1).

**Table 1:** Baseline characteristics of participants according to randomised treatment groups.

Characteristics	Randomised antihypertensive treatment	
	Placebo group	Active treatment group
No. of participants	2349	2343
Age, years	72.0 (6.7)	72.1 (6.7)
Female sex	1343 (57.2)	1320 (56.3)
Race, non-White	486 (20.7)	491 (21.0)
Education, years	11.7 (3.4)	11.7 (3.5)
Current smoking	300 (12.8)	292 (12.5)
Current drinking	695 (29.6)	703 (30.0)
History of stroke	31 (1.3)	35 (1.5)
History of MI	115 (4.9)	115 (4.9)
History of diabetes	241 (10.3)	235 (10.0)
CABG/PTCA	26 (1.1)	23 (1.0)
Angina^a^	119 (6.0)	137 (6.9)
SBP, mmHg	170.3 (9.2)	170.7 (9.5)
DBP, mmHg	76.6 (9.7)	77.0 (9.7)
Body mass index, kg/m^2^	27.5 (5.1)	27.5 (4.9)
Serum glucose, mmol/L	6.1 (1.9)	6.0 (1.9)
Cholesterol, mmol/L	6.1 (1.1)	6.1 (1.2)
HDL-C, mmol/L	1.4 (0.4)	1.4 (0.4)
Triglycerides, mmol/L	1.8 (1.1)	1.8 (1.1)
Depressive symptoms^b^	256 (11.0)	259 (11.1)

Note: Variables are mean (SD) or *n* (%).

CABG, coronary artery bypass grafting; PTCA, percutaneous transluminal coronary angioplasty; SBP, systolic blood pressure; DBP, diastolic blood pressure; HDL-C, high-density lipoprotein cholesterol.

^a^Angina measured by Rose questionnaire.

^b^Depressive symptom scale score of 7 or greater.

#### Association between baseline frailty index and dementia

**Figure 1:**
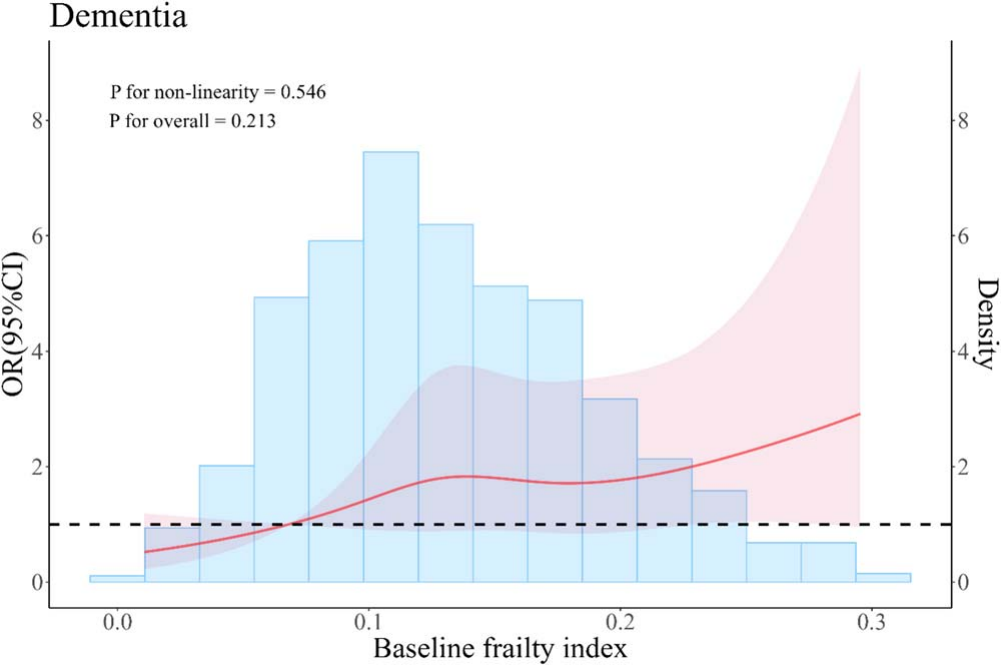
Association between baseline FI and dementia risk, with the distribution of FI.

During a median follow-up period of 4.4 years, 81 cases of dementia were recorded. Participants in the highest quarter of FI had an elevated risk of dementia compared to those in the lowest quarter, with an unadjusted OR of 3.49; 95% CI 1.73–7.05 (Table 2). However, this association was attenuated after adjusting for baseline age, sex, race, education and treatment group, with an adjusted OR (95% CI) of 1.91 (0.92–3.96) (Table 2). Baseline FI also showed a positive association with the risk of dementia, per SD increase in baseline FI (OR, 1.33; 95% CI, 1.08–1.64), with no evidence of a nonlinear relationship (*P* for nonlinearity >0.05; Figure 1).

**Table 2:** Association of baseline FI levels with dementia.

Frailty index	No. of events (%)	Unadjusted		Adjusted^b^	
		OR^a^ (95% CI)	*P*-value	OR^a^ (95% CI)	*P*-value
Categorical					
Quarter 1	11 (0.85)	Reference		Reference	
Quarter 2	15 (1.31)	1.58 (0.72–3.47)	0.248	1.33 (0.60–2.93)	0.482
Quarter 3	27 (2.29)	2.91 (1.44–5.90)	0.003	2.14 (1.04–4.40)	0.039
Quarter 4	28 (2.61)	3.49 (1.73–7.05)	<0.001	1.91 (0.92–3.96)	0.082
* P* for trend			<0.001		0.046
Continuous					
per SD increase	81 (1.73)	1.66 (1.36–2.02)	<0.001	1.33 (1.08–1.64)	0.007

Note: Quarter 1: FI ≤ 0.091; Quarter 2: 0.091 < FI ≤ 0.127; Quarter 3: 0.127 < FI ≤ 0.173; Quarter 4: FI > 0.173.

OR, odds ratio; CI: confidence interval.

^a^OR for risks of dementia according to quarters of or per SD increase in FI at baseline.

^b^Adjusted for age, race, sex, years of education and treatment group.

The association between baseline FI and the risk of dementia differed between treatment groups. In the active treatment group, per SD increase in FI was associated with a 57% higher risk of dementia (OR, 1.57; 95% CI, 1.16–2.11), after adjusting for baseline age, sex, race and years of education. However, this association was not statistically significant in the placebo group (1.17; 0.87–1.56); *P* for interaction = .049 (Table 3).

**Table 3:** Association between baseline FI and dementia according to randomised treatment groups.

Treatment group	No. of events (%)	Unadjusted	Adjusted^b^
		OR^a^ (95% CI)	OR^a^ (95% CI)
Active treatment	37 (1.58)	1.99 (1.51–2.63)	1.57 (1.16–2.11)
Placebo	44 (1.87)	1.39 (1.04–1.84)	1.17 (0.87–1.56)
*P* for interaction		0.073	0.049

OR, odds ratio; CI, confidence interval.

^a^OR for risks of dementia associated with per SD increase in FI at baseline.

^b^Adjusted for baseline age, sex, race, years of education.

#### Impact of baseline frailty levels on the efficacy of antihypertensive treatment for dementia

**Figure 2:**
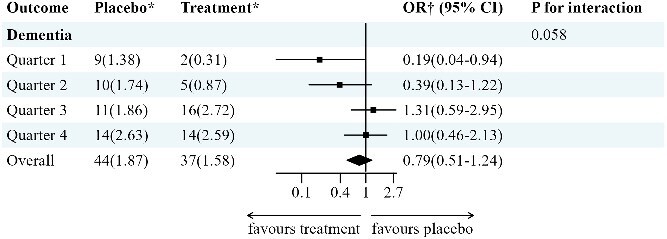
Randomised antihypertensive treatment effect on dementia according to the quarters of FI.

After adjusting for age, sex, race and years of education, randomised treatment was not significantly associated with a reduced risk of dementia (OR: 0.79; 95% CI: 0.51–1.24). Further adjustment for baseline FI did not alter the results (0.80; 0.51–1.25). However, in the lowest quarter of FI, antihypertensive treatment was associated with an 81% reduction in the risk of dementia (0.19; 0.04–0.94), after adjustments for baseline age, sex, race and education level (Figure 2). This protective effect was not observed in higher FI groups. A marginal significant interaction between treatment and FI groups was detected (*P* for interaction = .058, Figure 2).

When the FI was included as a continuous variable and explored graphically, antihypertensive treatment appeared to be associated with a lower risk of dementia only at lower FI levels, with a cut point of 0.200 being observed. However, no protective effect of antihypertensive treatment on dementia risk was observed in participants with FI above 0.200 (Appendix Figure S2).

#### Sensitivity analysis

We conducted a series of sensitivity analyses, which confirmed the robustness of our findings; full details are provided in the Appendix.

## Discussion

This post hoc analysis of the SHEP trial demonstrated that participants with isolated systolic hypertension with higher frailty levels were at a higher risk of dementia than less frail individuals. Furthermore, antihypertensive treatment exhibited a stronger protective effect against dementia in participants with lower frailty levels compared to those with higher frailty levels, suggesting that antihypertensive therapy may be more effective in reducing the risk of dementia in nonfrail populations.

### Frailty levels and dementia

Our results showed that baseline frailty level was associated with an increased risk of dementia in hypertensive participants aged over 60 years. Previous observational studies have identified similar findings of a strong association between higher frailty level and an increased risk of dementia [8, 22–24]. A pooled analysis of 29 849 adults aged ≥60 years from four cohorts showed that frailty trajectories accelerated 4–9 years before the onset of dementia [8]. These findings suggest that reverse causality is unlikely to explain the frailty–dementia association and that frailty may help identify individuals at high risk of dementia. However, evidence regarding the association between frailty and dementia in older individuals with hypertension remains limited. In line with our findings, the results of a post hoc analysis of SPRINT data, which included 8537 hypertensive patients, showed that higher baseline frailty levels were associated with an increased risk of cognitive impairment [14].

Our subgroup analysis revealed a borderline significant interaction between age groups and FI on the risk of dementia among SHEP participants. Frailty had a stronger effect on dementia in the younger group (<68 years) than the older subgroups (68–75 years and >75 years). When age was included as a continuous variable in the analysis, a significant interaction was observed between age and frailty levels. A longitudinal study found that individuals with a higher frailty level in middle age or earlier life were more likely to die before the age of 70, whereas those who lived past 80 tend to experience increases in frailty after age 70 [25]. This suggests that older frail participants in our study were less likely to have been frail since earlier adulthood and more likely to have developed frailty later in life. Consequently, individuals with the most severe frailty may have been underrepresented in the older age group due to higher pre-enrolment mortality, potentially leading to an underestimation of the impact of frailty in this population.

### Impact of baseline frailty levels on the efficacy of antihypertensive treatment for dementia

Several landmark antihypertensive trials have investigated the impact of antihypertensive treatment on dementia, but their findings have been inconsistent [26–29]. However, an individual participant data meta-analysis involving 28 008 participants from five similarly designed antihypertensive treatment trials (including SHEP) found that BP lowering was associated with a reduced risk of dementia [2]. This suggests that the inconsistent findings across individual trials may reflect variations in study design and differences in participants’ health profiles. Indeed, previous evidence has indicated that a patient’s health status may influence the effectiveness of antihypertensive treatment in preventing dementia [30]. Moreover, frailty, a critical syndrome already incorporated into hypertension management guidelines as a consideration for BP management strategies [5], may influence the effect of antihypertensive treatment on dementia risk. Evidence from observational studies suggested a potential impact of frailty on the association between BP and cognitive function [13, 31] and a possible interaction between frailty and antihypertensive treatment [32]. However, these studies are inevitably limited by their design and populations, which may include participants whose BP reduction reflects poor health or terminal decline, and thus may not accurately represent outcomes related to active BP control. Consequently, it is essential to explore the impact of frailty within the context of antihypertensive treatment trials. In our analysis of SHEP data, stratification by frailty levels revealed that antihypertensive treatment significantly reduced the risk of dementia in patients with lower frailty levels. Additionally, antihypertensive treatment modified the relationship between frailty and dementia, with the impact of frailty on dementia being more pronounced in the active treatment group compared to the placebo group. Notably, our subgroup analyses suggest that the frailty threshold at which antihypertensive treatment is no longer effective in preventing dementia may vary across different age groups. These findings are consistent with the results from the SPRINT data, which investigated a similarly aged population (mean age = 68 years). In SPRINT, intensive BP control was associated with a reduced risk of dementia among nonfrail participants, whereas this benefit was diminished in individuals with frailty. Additionally, within the intensive treatment group, the association between baseline frailty and an increased risk of dementia was stronger than in the standard treatment group [14].

Our findings can be attributed to the effect of antihypertensive treatment at lower FI levels, where reducing cardiovascular risk factors may delay the onset of dementia. This delay is likely because individuals in this group are in relatively good health and can benefit from BP reduction. However, for patients with higher frailty levels, antihypertensive treatment alone may be insufficient to mitigate the risk of dementia possibly due to the greater number of risk factors present in this subgroup. Patients with greater frailty may also have a reduced ability to maintain cerebral perfusion when BP levels vary thereby increasing the risk of cerebrovascular damage and functional impairment [33, 34]. However, hypertension is one of the leading risk factors for cardiovascular disease, and BP lowering in hypertensive patients is essential to reduce cardiovascular risk. Our findings therefore suggest that, in clinical practice, treatment strategies for hypertensive patients with higher levels of frailty should be combined with close monitoring of cognitive function to avoid additional cognitive risk. Combining cautious antihypertensive therapy with additional interventions aimed at reducing frailty, such as physical exercise [35, 36], nutritional supplementation [37, 38], might represent a more effective strategy for preventing dementia in this population.

### Strengths and limitations

This study utilised data from a multicentre, randomised, double-blind, placebo-controlled clinical trial, which provides more reliable results compared to observational studies. Further, compared to more recent trials, SHEP, as one of the earlier antihypertensive trials, enrolled participants with higher baseline BP levels, providing a greater potential for BP reduction [39]. This may help clarify whether the effectiveness of antihypertensive treatment is influenced by frailty status and extend our understanding to populations with higher average BP levels than those in SPRINT [40]. Moreover, benefiting from the comprehensive and rigorous data collection in the SHEP trial, our FI included 55 health deficits spanning multiple physiological systems, providing sufficient coverage to also construct an FI excluding cardiovascular-related factors.

There were some limitations that should be acknowledged. Firstly, as this was a post hoc analysis, we were unable to assess other frailty scores that require variables not collected in the SHEP trial. However, the 55-item FI used in this study provides a comprehensive assessment of frailty. Second, the antihypertensive strategy used in the SHEP trial, which primarily involved thiazide diuretics ± beta-blockers, may not align with contemporary treatment guidelines across various countries and regions, potentially limiting its applicability. However, a study analysing data from trials that used different antihypertensive treatment strategies also found similar results [14], suggesting that variation in antihypertensive drug class strategies is unlikely to influence the interaction effect we observed. Third, due to SHEP was a placebo-controlled trial, our analyses focused on the effects of antihypertensive treatment in frail individuals but could not directly inform optimal BP targets for this population. Determining appropriate BP targets in people with frailty remains an important question for future hypertension trials specifically designed for the frail group. Fourth, the findings of this study, particularly the subgroup analyses, may be affected by the limited number of dementia cases detected. Factors such as differing rates of dropout, mortality or cardiovascular events between treatment groups, along with the reduced follow-up period due to the trial’s early termination for cardiovascular benefits, constrained case observation [2, 41]. Therefore, future studies designed specifically to focus on dementia and with more dementia cases are required to validate our findings. Fifth, as with other clinical trials, our study may have excluded individuals who experienced greater frailty at a younger age and were either unable or unwilling to participate. This limitation could lead to an underestimation of the effects of frailty in older and more vulnerable populations. Finally, as SHEP trial was conducted in the 1980s–90s, cohort effects may limit generalisability, as participants’ sociodemographic profiles and disease burden may differ from those of contemporary populations [42].

## Conclusions

To our knowledge, this is the first study to report the impact of frailty status on the effects of antihypertensive treatment on dementia. Analysis of the SHEP trial data revealed that as frailty levels increased, the protective effect of antihypertensive treatment on dementia diminished. Our findings support that BP reduction in frail populations should be approached with caution, given its potential cognitive risks, and underscore the importance of frailty assessments in routine clinical practice. However, our study is limited by the generalizability of the clinical trial population and the insufficient statistical power due to dementia not being a primary outcome in antihypertensive trials. Therefore, we recommend future pooled analyses of antihypertensive trials with similar designs and have collected dementia outcomes to validate our findings.

## Supplementary Material

Supplementary_materials_afag037

## Data Availability

The data used in this article are available upon request from the NHLBI Biologic Specimen and Data Repository Information Coordinating Center (BioLINCC).
