# Supplementary material for: Association of frailty with dementia and the impact of frailty on antihypertensive treatment protection against dementia in older hypertensive adults
Source: Age Ageing. 2026 Feb 25;55(2):afag037. doi: 10.1093/ageing/afag037 (PMC13366261; doi:10.1093/ageing/afag037)
Supplement: Supplementary_materials_afag037 [file supplementary_materials_afag037.docx]

**Association of frailty with dementia and the impact of frailty on antihypertensive treatment protection against dementia** **in older hypertensive adults**

**Appendix:**

[Frailty index 3](#_Toc219973248)

[Results of sensitivity analysis 4](#_Toc219973249)

[Table S1. Items included in baseline frailty index (FI) 5](#_Toc219973250)

[Table S2. Subgroup analysis of the association between baseline FI and dementia 9](#_Toc219973251)

[Table S3. Association between dementia risk and FI categories based on different cut-off values. 10](#_Toc219973252)

[Table S4. Randomized antihypertensive treatment effect on dementia according to FI categories. 11](#_Toc219973253)

[Table S5. Association between baseline FI without CVD related deficits and dementia 12](#_Toc219973254)

[Table S6. Effect of randomized antihypertensive treatment on dementia according to median (0.121) of FI without CVD deficits 13](#_Toc219973255)

[Figure S1. Distribution of baseline FI in randomized treatment groups 14](#_Toc219973256)

[Figure S2. Effects of randomized antihypertensive treatment on dementia according to baseline FI level 15](#_Toc219973257)

[Figure S3 Subgroup analysis of randomized antihypertensive treatment effect on dementia according to baseline FI level 16](#_Toc219973258)

[Figure S4. Randomized antihypertensive treatment effect on dementia according to the quarters of FI without cardiovascular-related factors 17](#_Toc219973259)

[Reference 18](#_Toc219973260)

# Frailty index

Baseline frailty levels of participants were quantified using a frailty index (FI) developed following the standardized methodology (1), whereby a person’s symptoms, signs, diseases, and disabilities are considered as deficits, which are combined as an FI. To be eligible for inclusion in the FI, deficits must be: (ⅰ) health-related; (ⅱ) associated with age; (ⅲ) < 5% missing values; (ⅳ) neither overly rare (deficit present in less than 1% of the study population), nor overly common (deficit present in more than 80% of the population under study). In the baseline data of SHEP, we identified 55 suitable variables, including lifestyle, medical history, medication history, physical examination, electrocardiogram, laboratory tests, behavioral and cognitive factors. These variables were used to establish the FI for the study participants (Appendix Table S1). To construct the FI, each variable was recoded on a scale from 0 to 1, where 0 indicated the absence of a deficit and 1 indicated its full presence. In accordance with standard FI methodology, we did not impute or exclude missing values within individual variables. For each participant, the FI was calculated as the sum of all deficit scores divided by the number of available variables for that participant. FI values range from 0 (absence of any deficits) to 1 (presence of all deficits), but a submaximal limit exists in practice, with 99% of individuals having FI values below 0.7. Individuals with FI values exceeding 0.7 are rarely observed due to higher mortality rates (2, 3). An FI was not calculated for 44 participants missing more than 11 variables (20% of the deficits included in FI) and those participants were excluded from the analysis**.** FI was categorized into quarters, with higher quarters representing greater levels of frailty.

# Results of sensitivity analysis

In participants younger than 68 years, each SD increase in FI was associated with a higher risk of dementia (OR: 2.41, 95% CI: 1.07–5.46, Appendix Table S2). In those aged 68–75 years, the association was not statistically significant (1.51, 0.95–2.42). In participants older than 75 years, FI was associated with a higher dementia risk (1.28, 1.01–1.63). Overall, the interaction between age group and FI on the risk of dementia was not statistically significant (*P* = 0.081, Appendix Table S2). However, an interaction analysis using age as continuous variable and FI revealed a significant interaction between the two variables (*P* = 0.018). The association between FI and dementia was consistent by sex, education levels, baseline SBP (all *P* for interaction > 0.05, Appendix Table S2). Stratified analysis by age, gender, education, and SBP also showed a consistent decline in treatment efficacy with increasing FI across all subgroups (Appendix Figure S3). Further, Participants were regrouped using alternative FI thresholds, including the FI median (FI = 0.127), FI = 0.200, a cut-point of 0.21, and a three-level categorization of fit (FI ≤0.12), mild frailty (FI >0.12–0.24), and moderate to severe frailty (FI >0.24). Analyses based on these alternative groupings yielded results that were consistent with our main findings (Appendix Table S3 and Appendix Table S4). Finally, higher levels of the revised FI, excluding cardiovascular-related factors remained significantly associated with increased dementia risk (Appendix Table S5), with the protective effect of antihypertensive treatment remaining more pronounced among participants with lower as compared higher scores revised FI scores (Appendix Table S6 and Appendix Figure S4).

# Table S1. Items included in baseline frailty index (FI)

| Items | | Scoring (0: absence of a deficit, 1: the full deficit) | Deficits  n (%) | Missing  n (%) | Note |
| --- | --- | --- | --- | --- | --- |
| Lifestyle | | | | | |
| 1 | Current smoking | No: 0 Yes: 1 | 602(12.7) | 2(0.04) |  |
| 2 | Drinking | <1 drink / week: 0 ≥ 1 drink / week:1 | 1409(29.7) | 0(0) |  |
| 3 | Living alone | No: 0 Yes: 1 | 1586(33.9) | 65(1.4) |  |
| Physical examination | | | | | |
| 4 | Pulse rate | 60-100 bpm:0 <60 or >100 bpm: 1 | 547(11.5) | 4(0.8) |  |
| 5 | SBP | < 175: 0 ≥ 175: 1 | 1238(26.1) | 0(0) | Use the cut-off point of 75th percentile |
| 6 | DBP | < 83.5: 0 ≥83.5: 1 | 1207(25.5) | 0(0) | Use the cut-off point of 75th percentile |
| 7 | BMI | BMI≥18.5 and <25(white)/23.4(black): 0  BMI≥25 (white)/23.4(black) and <30(white)/28.1(black): 0.5 BMI≥30 (white)/28.1(black) or <18.5: 1 | 0.5: 1994(42.6)  1: 1302(27.8) | 58(1.2) | Cut-off: Ethnicity-specific BMI cut-off for obesity based on type 2 diabetes risk in England: a population-based cohort study |
| Medicine and medical history | | | | | |
| 8 | Polypharmacy | <5 drugs: 0 ≥5 drugs: 1 | 684(14.5) | 6(0.1) |  |
| 9 | History of stroke or TIA | No: 0 Yes: 1 | 73(1.5) | 18(0.4) | Combine history of stroke and TIA |
| 10 | History of MI or angina | No: 0  Angina: 0.5 MI: 1 | 0.5:222(4.7)  1.0:232(4.9) | 13(0.3) | Combine MI and angina |
| 11 | CABG/PTCA | No: 0 Yes: 1 | 49(1.0) | 98(2.1) | Combine angioplasty and coronary bypass |
| 12 | History of Diabetes | No: 0 Yes: 1 | 478(10.1) | 2(0.04) |  |
| 13 | History of Chronic Bronchitis | No: 0 Yes: 1 | 148(3.1) | 12(0.2) |  |
| 14 | History of Emphysema | No: 0 Yes: 1 | 122(2.6) | 15(0.3) |  |
| 15 | History of Fractures | No: 0 Yes: 1 | 478(10.1) | 11(0.2) | Combine hip, spine, forearm fractures |
| 16 | History of memory or other brain problem | No: 0 Yes: 1 | 209(4.4) | 2(0.04) |  |
| 17 | History of Gout | No: 0 Yes: 1 | 359(7.6) | 2(0.04) |  |
| 18 | History of Cancer | No: 0 Yes: 1 | 434(9.2) | 3(0.06) |  |
| 19 | History of Falls | < 2 times in past three months: 0 ≥2 times in past three months: 1 | 49(1.0) | 2(0.04) |  |
| 20 | Rose Questionnaire intermittent claudication | No: 0 Yes: 1 | 111(2.3) | 2(0.04) |  |
| Laboratory | | | | | |
| 21 | Sodium | 134-143mmol/L: 0 <134 or >143mmol/L: 1 | 277(6.1) | 230(4.8) | Cut-off from SHEP central laboratory METPATH |
| 22 | Creatinine | 53.0-114.9 μmol/L: 0 <53.0 or >114.9 μmol/L: 1 | 750(16.6) | 232(4.9) | Cut-off from SHEP central laboratory METPATH |
| 23 | Blood urea nitrogen | 2.1-8.2mmol/L: 0 <2.1 or >8.2mmol/L: 1 | 382(8.5) | 224(4.7) | Cut-off from SHEP central laboratory METPATH |
| 24 | Calcium | 2.2-2.6mmol/L: 0 <2.2 or >2.6mmol/L: 1 | 138(3.1) | 233(4.9) | Cut-off from SHEP central laboratory METPATH |
| 25 | Serum potassium | 3.5-5.3mmol/L: 0 <3.5 or >5.3mmol/L: 1 | 196(4.3) | 196(4.1) | Cut-off from SHEP central laboratory METPATH |
| 26 | Glucose | 3.6-7.2mmol/L: 0 <3.6 or >7.2mmol/L: 1 | 546(12.1) | 223(4.7) | Cut-off from SHEP central laboratory METPATH |
| 27 | Alkaline phosphatase | 10-45 U/L: 0 <10 or >45 U/L: 1 | 249(5.5) | 232(4.9) | Cut-off from SHEP central laboratory METPATH |
| 28 | Serum glutamic-oxaloacetic transaminase | 10-50 U/L: 0 <10 or >50 U/L: 1 | 125(2.8) | 229(4.8) | Cut-off from SHEP central laboratory METPATH |
| ECG | | | | | |
| 29 | Left ventricular hypertrophy | No: 0 Yes: 1 | 348(7.4) | 65(1.4) |  |
| 30 | Ventricular conduction defects | No: 0 Yes: 1 | 408(8.7) | 65(1.4) |  |
| 31 | A-V conduction defects | No: 0 Yes: 1 | 226(4.8) | 65(1.4) |  |
| 32 | Premature beats | No: 0 Yes: 1 | 881(19.0) | 91(1.9) |  |
| 33 | Q/QS patterns abnormalities | No: 0 Yes: 1 | 341(7.3) | 65(1.4) |  |
| 34 | Left QRS axis deviation | No: 0 Yes: 1 | 572(12.2) | 69(1.4) |  |
| Behavioral variables | | | | | |
| 35 | SHROT-CARE Depressive Symptoms Scores | <7: 0 ≥7: 1 | 521(11.0) | 24(0.5) |  |
| 36 | Activities of Daily Living Scores | =7: 0 <7: 1 | 253(5.4) | 34(0.7) |  |
| 37 | Heavy work around the house | Able:0 Unable:1 | 435(9.3) | 70(1.4) |  |
| 38 | Walk up and down stairs | Able:0 Unable:1 | 103(2.2) | 57(1.2) |  |
| 39 | Walk half a mile without help | Able:0 Unable:1 | 253(5.4) | 102(2.1) |  |
| 40 | Pulling or pushing large objects | No difficulty:0 A little difficulty:0.25 Some difficulty:0.50 A lot of difficulty:0.75 Unable:1.00 | 0.25: 604(12.9) 0.50: 305(6.5) 0.75: 95(2.0) 1.00: 115(2.4) | 47(0.1) |  |
| 41 | Stooping, crouching or kneeling | No difficulty:0 A little difficulty:0.25 Some difficulty:0.50 A lot of difficulty:0.75 Unable:1.00 | 0.25: 969(20.6) 0.50: 514(10.9) 0.75: 263(5.6) 1.00: 147(3.1) | 37(0.8) |  |
| 42 | Reaching or extending arms above shoulder level | No difficulty:0 A little difficulty:0.25 Some difficulty:0.50 A lot of difficulty:0.75 Unable:1.00 | 0.25: 220(4.7) 0.50: 113(2.4) 0.75: 43(0.9) 1.00: 29(0.6) | 32(0.7) |  |
| 43 | Lifting or carrying weights under 10 pounds | No difficulty:0 A little difficulty:0.25 Some difficulty:0.50 A lot of difficulty:0.75 Unable:1.00 | 0.25: 228(4.8) 0.50: 116(2.5) 0.75: 62(1.3) 1.00: 60(1.3) | 37(0.8) |  |
| 44 | Writing or handling small objects | No difficulty:0 A little difficulty:0.25 Some difficulty:0.50 A lot of difficulty:0.75 Unable:1.00 | 0.25: 288(6.1) 0.50: 101(2.1) 0.75: 35(0.7) 1.00: 0(0) | 32(0.7) |  |
| 45 | Social-network: marital status | Yes: 0 No:1 | 237(5.1) | 135(2.8) | Yes: include Married widowed, divorced, separated; No: Never married |
| 46 | Social-network: children number | ≥1:0 0:1 | 880(19.1) | 137(2.9) |  |
| 47 | Social-network: number of children see at least once a week | ≥1:0 0:1 | 2191(47.6) | 137(2.9) |  |
| 48 | Social-network: number of children talk to on phone or correspond at least once a week | ≥1:0 0:1 | 1481(32.2) | 139(2.9) |  |
| 49 | Social-network: number of close relatives | ≥1:0 0:1 | 1012(22.0) | 138(2.9) |  |
| 50 | Social-network: number of relatives see at least once a month | ≥1:0 0:1 | 2377(51.7) | 140(2.9) |  |
| 51 | Social-network: number of relatives correspond with by phone or letter at least once a month | ≥1:0 0:1 | 1102(24.0) | 138(2.9) |  |
| 52 | Social-network number of close friends | ≥1:0 0:1 | 633(13.8) | 157(3.3) |  |
| 53 | Social-network: number of friends see at least once a month | ≥1:0 0:1 | 887(19.4) | 157(3.3) |  |
| 54 | Social-network: number of friends correspond with by phone or letter at least once a month | ≥1:0 0:1 | 774(16.9) | 160(3.4) |  |
| 55 | Social-network: involved in any groups | Yes: 0 No: 1 | 1332(29.0) | 139(2.9) |  |
| SBP: Systolic blood pressure; DBP: diastolic blood pressure; BMI: body mass index; TIA: transient ischemic attack; MI: myocardial infarction; CABG: coronary-artery bypass grafting; PTCA: percutaneous transluminal coronary angioplasty. | | | | | |

# Table S2. Subgroup analysis of the association between baseline FI and dementia

| Subgroups | OR^*^ (95%CI) | *P* for interaction |
| --- | --- | --- |
| Age thirds |  | 0.081 |
| < 68 years | 2.41(1.07-5.46) |  |
| 68 - 75 years | 1.51(0.95-2.42) |  |
| > 75 years | 1.28(1.01-1.63) |  |
|  |  |  |
| Sex |  | 0.322 |
| Female | 1.30(1.02-1.66) |  |
| Male | 1.49(0.98-2.28) |  |
|  |  |  |
| Education |  | 0.324 |
| < 12 years | 1.21(0.89-1.63) |  |
| ≥ 12 years | 1.44(1.09-1.91) |  |
|  |  |  |
| SBP thirds |  | 0.445 |
| < 165 mmHg | 1.06(0.68-1.66) |  |
| 165 - 173 mmHg | 1.86(1.27-2.74) |  |
| > 173 mmHg | 1.13(0.82-1.57) |  |
| * OR for risks of dementia associated with per SD increase in frailty index at baseline, adjusted for baseline age, gender, race, years of education and treatment group, unless used as grouping variables.  CI: confidence interval; OR: Odds ratio; SBP: systolic blood pressure. | | |

# Table S3. Association between dementia risk and FI categories based on different cut-off values.

| Frailty index | No. of events (%) | Unadjusted | | Adjusted* | |
| --- | --- | --- | --- | --- | --- |
|  |  | OR ^†^ (95%CI) | *P*-value | OR^†^ (95%CI) | *P*-value |
| FI < 0.127 | 23(1.02) | Reference |  | Reference |  |
| FI ≥ 0.127 | 58(2.38) | 1.58(0.72-3.47) | <0.001 | 1.77(1.07-2.94) | 0.026 |
|  |  |  |  |  |  |
| FI < 0.200 | 58(1.45) | Reference |  | Reference |  |
| FI ≥ 0.200 | 23(3.35) | 2.61(1.60-4.27) | <0.001 | 1.63(0.97-2.74) | 0.062 |
|  |  |  |  |  |  |
| FI < 0.210 | 63(1.51) | Reference |  | Reference |  |
| FI ≥ 0.210 | 18(3.44) | 2.63(1.54-4.48) | <0.001 | 1.59(0.91-2.78) | 0.105 |
|  |  |  |  |  |  |
| FI ≤ 0.120 | 21(0.97) | Reference |  | Reference |  |
| FI > 0.12-0.24 | 47(2.09) | 2.30(1.37-3.87) | 0.002 | 1.66(0.97-2.83) | 0.064 |
| FI > 0.24 | 13(4.47) | 5.67(2.80-11.48) | <0.001 | 2.96(1.41-6.22) | 0.004 |
| *Adjusted for age, race, sex, years of education and treatment group.  † OR for the risks of dementia in the higher FI group compared to the lower FI group.  OR: odds ratio; CI: confidence interval. | | | | | |

# Table S4. Randomized antihypertensive treatment effect on dementia according to FI categories.

| FI group | No. of events (%) | | OR* (95%CI) | *P* for interaction |
| --- | --- | --- | --- | --- |
|  | Placebo | Treatment |  |  |
| FI < 0.127 | 18(1.60) | 5(0.4) | 0.23(0.08-0.65) | 0.007 |
| FI ≥ 0.127 | 26(2.12) | 32(2.65) | 1.22(0.71-2.08) |  |
|  |  |  |  |  |
| FI < 0.200 | 33(1.64) | 25(1.25) | 0.69(0.41-1.18) | 0.343 |
| FI ≥ 0.200 | 11(3.26) | 12(3.43) | 1.18(0.50-2.77) |  |
|  |  |  |  |  |
| FI < 0.210 | 37(1.77) | 26(1.25) | 0.64(0.38-1.06) | 0.065 |
| FI ≥ 0.210 | 7(2.69) | 11(4.18) | 1.91(0.70-5.25) |  |
|  |  |  |  |  |
| FI ≤ 0.120 | 16(1.49) | 5(0.46) | 0.29(0.10-0.81) | 0.005 |
| FI > 0.12-0.24 | 24(2.13) | 23(2.06) | 0.88(0.49-1.59) |  |
| FI > 0.24 | 4(2.74) | 9(6.34) | 3.13(0.86-11.39) |  |
| * OR for treatment effect on dementia, adjusted for age, sex, race, years of education at baseline  OR: Odds ratio; CI: Confidence interval. | | | | |

# Table S5. Association between baseline FI without CVD related deficits and dementia

| Frailty index | No. of events (%) | Unadjusted | | Adjusted* | |
| --- | --- | --- | --- | --- | --- |
|  |  | OR ^†^ (95%CI) | *P*-value | OR^†^ (95%CI) | *P*-value |
| Categorical |  |  |  |  |  |
| Quarter 1 | 10(0.85) | Reference |  | Reference |  |
| Quarter 2 | 13(1.15) | 1.38(0.60-3.15) | 0.451 | 1.04(0.45-2.41) | 0.929 |
| Quarter 3 | 28(2.41) | 3.01(1.45-6.22) | 0.003 | 1.92(0.91-4.05) | 0.087 |
| Quarter 4 | 27(2.46) | 3.26(1.57-6.76) | 0.002 | 1.52(0.70-3.26) | 0.287 |
| *P* for trend |  |  | <0.001 |  | 0.154 |
| Continuous |  |  |  |  |  |
| per SD increase | 78(1.71) | 1.62(1.33-1.96) | <0.001 | 1.25(1.01-1.55) | 0.044 |
| Quarter 1: FI ≤ 0.071; Quarter 2: 0.071 < FI ≤ 0.121; Quarter 3: 0.121 < FI ≤ 0.186; Quarter 4: FI > 0.186.  * Adjusted for age, race, sex, years of education and treatment group.  † OR for risks of dementia according to quarters of or per SD increase in FI at baseline.  OR: odds ratio; CI: confidence interval.  Cardiovascular-related factors excluded from the original FI including current smoking, alcohol consumption, pulse rate, systolic and diastolic blood pressure, body mass index, history of diabetes, history of stroke or transient ischemic attack, history of myocardial infarction or angina, coronary artery bypass grafting/percutaneous transluminal coronary angioplasty, all electrocardiogram variables, and serum levels of sodium, glucose, potassium, and calcium | | | | | |

# Table S6. Effect of randomized antihypertensive treatment on dementia according to median (0.121) of FI without CVD deficits

| FI group | No. of events (%) | | OR* (95%CI) | *P* for interaction |
| --- | --- | --- | --- | --- |
|  | Placebo | Treatment |  |  |
| FI < 0.121 | 17(1.56) | 5(0.46) | 0.24(0.08-0.68) | 0.018 |
| FI ≥ 0.121 | 26(2.17) | 30(2.51) | 1.10(0.64-1.89) |  |
| * OR for treatment effect on dementia, adjusted for age, sex, race, years of education at baseline  OR: Odds ratio; CI: Confidence interval.  Cardiovascular-related factors excluded from the original FI including current smoking, alcohol consumption, pulse rate, systolic and diastolic blood pressure, body mass index, history of diabetes, history of stroke or transient ischemic attack, history of myocardial infarction or angina, coronary artery bypass grafting/percutaneous transluminal coronary angioplasty, all electrocardiogram variables, and serum levels of sodium, glucose, potassium, and calcium. | | | | |

# Figure S1. Distribution of baseline FI in randomized treatment groups


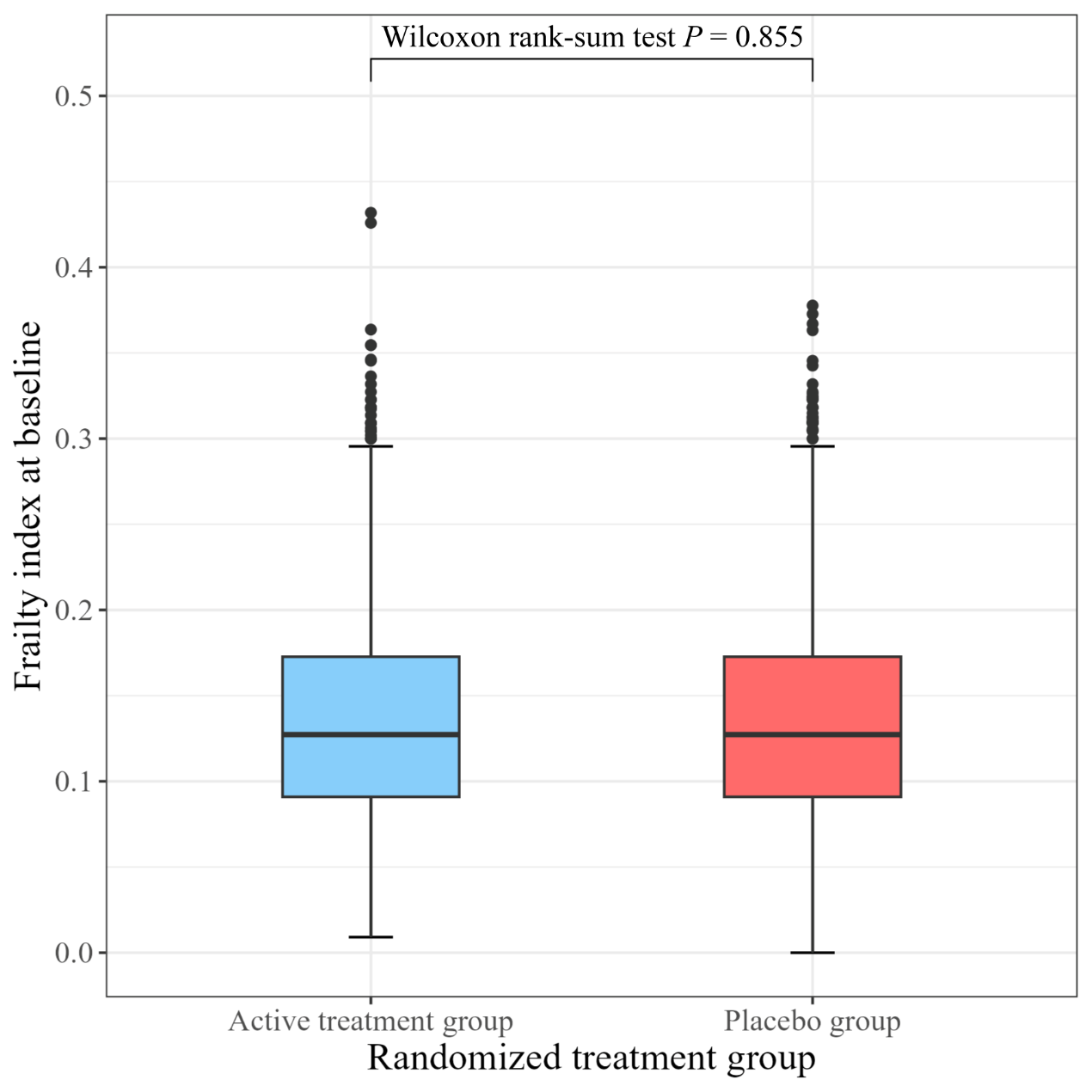


# Figure S2. Effects of randomized antihypertensive treatment on dementia according to baseline FI level


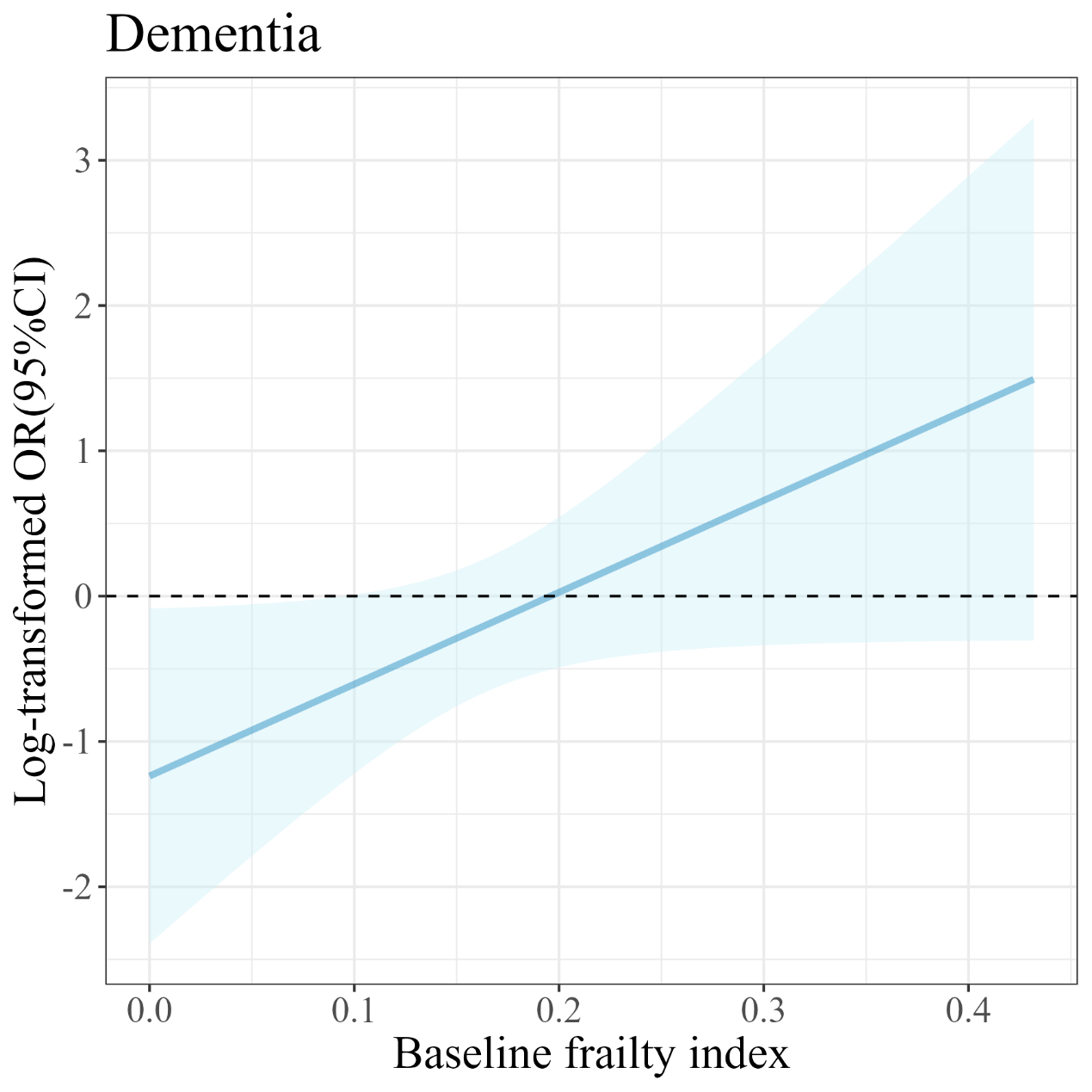


The blue line and shade represent odds ratios and 95% CIs for treatment effect on dementia derived from conditional comparisons based on logistic model adjusted for age, sex, race, years of education.

OR: odds ratio; CI: confidence interval;

# Figure S3 Subgroup analysis of randomized antihypertensive treatment effect on dementia according to baseline FI level


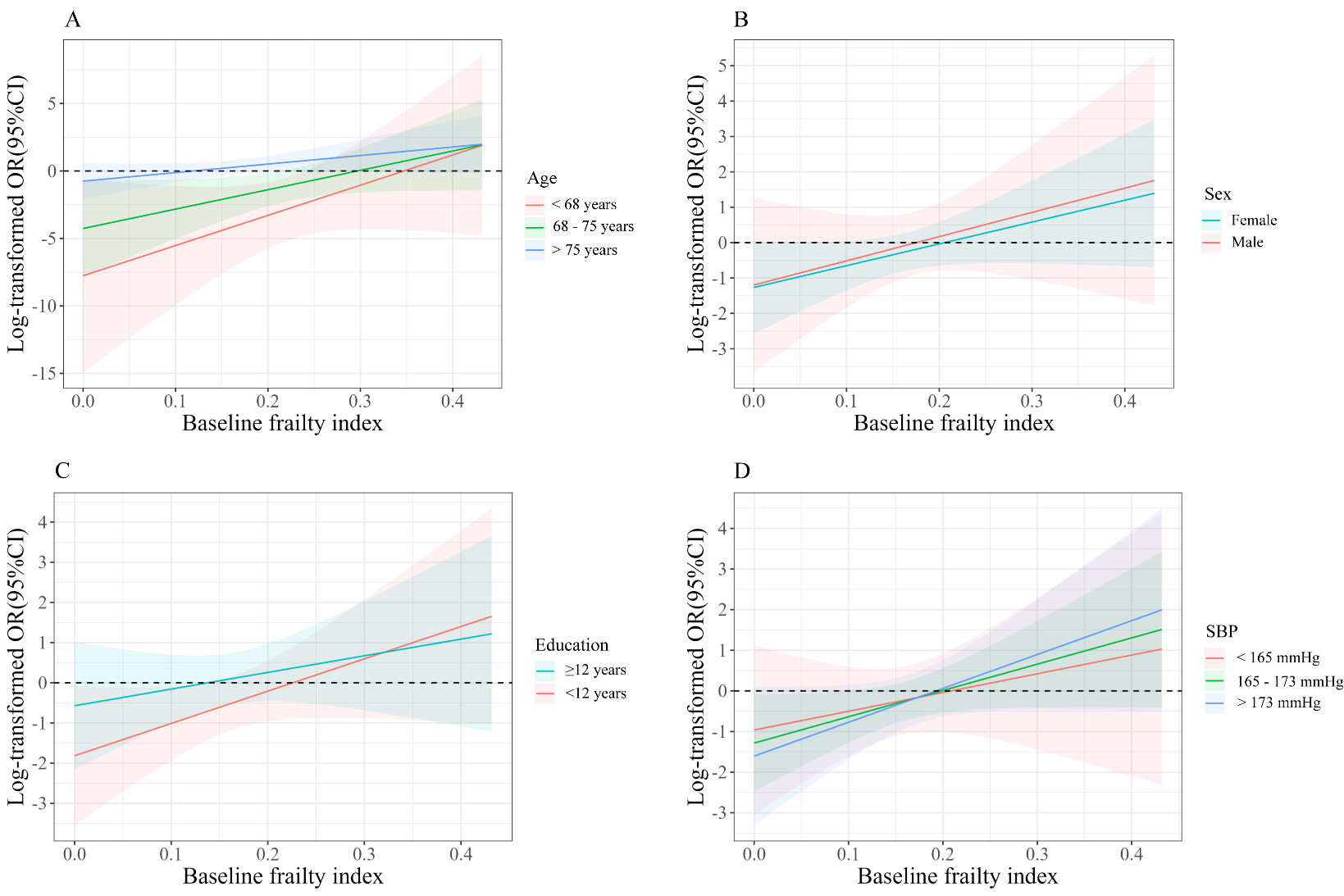


A: age subgroup; B: sex subgroup; C education subgroup; D: SBP subgroup.

OR for treatment effect on dementia, adjusting for age, sex, race and years of education, unless used as grouping variables.

OR: odds ratio; CI: confidence interval; SBP: systolic blood pressure.

# Figure S4. Randomized antihypertensive treatment effect on dementia according to the quarters of FI without cardiovascular-related factors


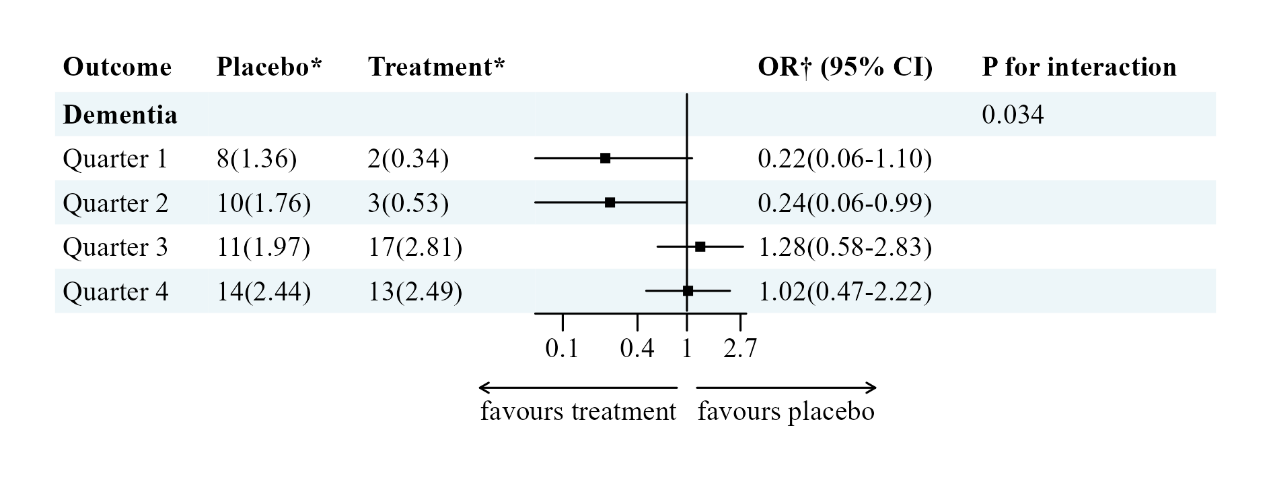


Quarter 1: FI ≤ 0.071; Quarter 2: 0.071 < FI ≤ 0.121; Quarter 3: 0.121 < FI ≤ 0.186; Quarter 4: FI > 0.186.

*Number of events and their percentage of treatment groups, according to FI groups.

† OR for treatment effect on outcome events, adjusting for age, sex, race and years of education.

Cardiovascular-related factors excluded from the original FI including current smoking, alcohol consumption, pulse rate, systolic and diastolic blood pressure, body mass index, history of diabetes, history of stroke or transient ischemic attack, history of myocardial infarction or angina, coronary artery bypass grafting/percutaneous transluminal coronary angioplasty, all electrocardiogram variables, and serum levels of sodium, glucose, potassium, and calcium.

# Reference

1. Theou O, Haviva C, Wallace L, et al. How to construct a frailty index from an existing dataset in 10 steps. Age Ageing. 2023;52(12).

2. Rockwood K, Mitnitski A. Limits to deficit accumulation in elderly people. Mech Ageing Dev. 2006;127(5):494-6.

3. Rockwood K, Rockwood MR, Mitnitski A. Physiological redundancy in older adults in relation to the change with age in the slope of a frailty index. J Am Geriatr Soc. 2010;58(2):318-23.
